# Mixed silage with Chinese cabbage waste enhances antioxidant ability by increasing ascorbate and aldarate metabolism through rumen *Prevotellaceae UCG-004* in Hu sheep

**DOI:** 10.3389/fmicb.2022.978940

**Published:** 2022-08-26

**Authors:** Chuang Li, Ning Chen, Xingxing Zhang, Khuram Shahzad, Ruxin Qi, Zhenbin Zhang, Zhiqi Lu, Yue Lu, Xiang Yu, Muhammad Hammad Zafar, Mengzhi Wang, Wujun Liu

**Affiliations:** ^1^College of Animal Science and Technology, Yangzhou University, Yangzhou, China; ^2^State Key Laboratory for Sheep Genetic Improvement and Healthy Production, Xinjiang Academy of Agricultural and Reclamation Science, Shihezi, China; ^3^Institute of Animal Husbandry and Veterinary, Xinjiang Academy of Agricultural and Reclamation Science, Shihezi, China; ^4^Department of Biosciences, COMSATS University Islamabad, Islamabad, Pakistan; ^5^College of Animal Science, Xinjiang Agricultural University, Urumqi, China

**Keywords:** mixed silage, Hu sheep, antioxidant properties, high-throughput sequencing, bacterial community

## Abstract

Silage is rich in nutrients, which can make up for the lack of seasonal roughage, and has a certain promotion effect on the intensive feeding of ruminants. In addition, silage can maintain the rumen function of ruminants to a certain extent and reduce the risk of rumen acidosis and abomasum translocation. The purpose of this study was to investigate the effects of the mixed silage of Chinese cabbage waste and rice straw (mixed silage) on antioxidant performance, rumen microbial population, and fermentation metabolism of Hu sheep. The 16 healthy Hu sheep (eight rams and eight ewes, 39.11 ± 1.16 kg, 5.5 months) were randomly divided into two groups (the control group and the mixed silage group) with eight animals (four rams and four ewes) in each group. The control group was fed with farm roughage (peanut seedlings, corn husk, and high grain shell) as forage, and the mixed silage group was fed with the mixed silage as forage. The results showed that the mixed silage had no effect on the growth performance of Hu sheep (*p* > 0.05). Ruminal butyric acid, total volatile fatty acids (TVFA), and ammonia nitrogen (NH_3_-N) concentration in the mixed silage group were increased, whereas the pH was decreased (*p* < 0.05). The blood and rumen total antioxidants capacity (T-AOC) concentration in the mixed silage group was higher, and the malondialdehyde (MDA) content in rumen, serum, liver, and kidney was lower than that in the control group (*p* < 0.05). PCoA and ANOSIM results of Illumina sequencing indicated that the mixed silage affected the bacterial composition of the rumen microbes. The mixed silage increased the proportion of *Prevotellaceae UCG-004* which was in a positive correlation with Vitamin C (Vc). In addition, PICRUSt functional prediction analysis showed that ascorbate and aldarate metabolism were up-regulated in the mixed silage group (*p* < 0.05). In conclusion, higher contents of V_C_ and acid detergent fiber (ADF) in the mixed silage were beneficial to the growth and reproduction of *Prevotellaceae UCG-004*, resulting in increased production of the butyric acid significantly upregulated the metabolism of ascorbate and aldarate metabolism, thereby improving the antioxidant properties of Hu sheep.

## Introduction

As the global population increases, so does the demand for animal products. The growing demand for animal products has also created a huge requirement for animal feed, and the efficient use of existing feed is essential for efficient livestock farming and food safety (Makkar and Ankers, 2014). Chinese vegetable production accounts for 50% of the world’s total production (FAOSTAT, 2016), but it is also accompanied by a large amount of vegetable waste. Vegetable waste, such as cabbage tails, accounts for the majority of food waste, especially in supermarkets, fresh food markets and households (Ma et al., 2017). The rational use of vegetable waste can not only reduce environmental pollution (water and soil pollution, bacteria reproduction, etc.) but also reduce the harm to consumers’ health (Qiao et al., 2020). In addition, vegetable tails contains minerals, vitamins and other nutrients, which can promote the antioxidant properties of ruminants (Seong et al., 2016; Sahoo et al., 2021). The annual production of rice straw in China is about 21 million t/year, accounting for about 47% of the straw production (Wang et al., 2010; Chen, 2016). Fresh crop stalks dry and wilt rapidly, resulting in reduced nutrients such as moisture and soluble substances (Liu et al., 2019). Furthermore, The lower nutritional value and higher silica content of straw results in lower feed utilization and digestibility of rice straw (Cherdthong et al., 2020; Suntara et al., 2020).

There is a certain complementary effect between Chinese cabbage waste and rice straw in terms of physical structure, nutrients, and water content. Air-dried straw stalks and Chinese cabbage waste are available to complete common silage (Ren et al., 2018). Previous studies showed that vegetable waste can be mixed with straw for silage, leading to the higher rate of utilization, for example, broccoli by-products were mixed with wheat straw (Partovi et al., 2020); corn stover were mixed with cabbage (Ren et al., 2018), co-ensiling of sugar beet waste, and wheat straw (Hillion et al., 2018).

Silage treatment can be used for feed utilization of unconventional feed, and has certain positive impacts on the health status, physiology and rumen microorganisms of ruminants (Bampidis and Robinson, 2006; Abd El Tawab et al., 2020). Broccoli by-product and wheat straw (ratio 69:31) mixed feed did not affect rumen fermentation parameters in Fasander lambs (Partovi et al., 2020). In addition, adding 10% silage mulberry leaves to the total mixed diet of Hanwoo cattle can improve the activities of antioxidant enzymes such as total superoxide dismutase (T-SOD) and glutathione peroxidase (GSH-Px; Cheong et al., 2012). Hassanat et al. (2013) found that by increasing the proportion of maize silage in the diet, the richness and diversity of bacterial communities was decreased, but the number of total bacteria and *Prevotella* spp., was increased which favored the propionate production.

The rumen environment and microflora structure are crucial to the digestion and absorption of nutrients, production performance, and health. The rumen is home to a large number of microorganisms that grow in an anaerobic environment and can adhere to the feed surface and ferment (Palma-Hidalgo et al., 2021). Diet level, geographic distribution, feeding management, and health status are several factors that affect the rumen environment and microbial community structure while the diet level is the biggest factor affecting rumen fermentation and microbial population changes (Henderson et al., 2015; van Lingen et al., 2016).

We hypothesized that the mixed silage could improve the rumen environment and microbial flora structure, thereby enhancing the antioxidant properties of Hu sheep. Therefore, in the present study, we fed Hu sheep mixed silage to explore its effects on the antioxidant properties, rumen fermentation and microorganisms, so as to provide some reference for the application of mixed silage in Hu sheep.

## Materials and methods

### Mixed silage source and production process

The rice straw was collected from the experimental farms of Yangzhou University in Jiangsu Province. The Chinese cabbage waste was collected from the East Garden Farmers Market in Yangzhou City, Jiangsu Province, China. Lactobacillus plantarum and cellulase used in the experiment were purchased from Guangzhou Lvhui Biotechnology Co., Ltd.

The rice straw and Chinese cabbage tails were cut to 2–3 cm, and then were mixed thoroughly with *Lactobacillus plantarum* and *cellulase* in a ratio of 4:6. *Lactobacillus plantarum* 0.035 g/kg and *cellulase* 0.25 g/kg were added to each kilogram of mixed silage. After mixing, it was put into silage bags, vacuum-treated with a Meggis vacuum machine, sealed in a cool place for 45 days, and then silaged and sampled after anaerobic fermentation.

### Determination of vitamin content

After the mixed silage was completed, the contents of vitamin A (V_A_), vitamin B_2_ (V_B2_), V_C_, and vitamin E (V_E_) were determined by commercial kits (Shanghai Enzyme Link Biotechnology Co., Ltd.).

### Experimental animals and feeding

We selected 16 healthy Hu sheep with an initial weight of 39.97 ± 1.22 kg and an age of about 5.5 months, and randomly divided them into two groups (*n* = 8, four rams and four ewes). We took the sheep farm roughage (peanut seedlings, corn husks and high grain husks) as the control group, and the mixed silage group took the mixed silage as the roughage. Both the control group and the mixed silage group were fed 50% roughage and 50% concentrate (based on dry matter (DM), Table 1). Feed formulations were designed according to the nutritional requirements of mutton sheep (NRC, 2007). The feeding experiment was carried out for a total of 5 weeks. The first week was the pre-feeding period, and the remaining period from the second to the fifth week was the experimental period. The experimental Hu sheep were raised in single pens, and the sheep house was cleaned and disinfected before the experiment. All sheep were uniformly dewormed and immunized before entering into the sheep house. In the pre-feeding period and the formal experimental period, each Hu sheep was fed twice a day (8:00 and 18:00). During the experiment, each Hu sheep was free to get clean drinking water.

**Table 1:** Experimental diet formula and nutrition level (DM basis).

Item	Groups^1^	
	Control	Mixed silage
Ingredients (% of DM)		
Peanut seedling	30.00	–
Corn husk	15.00	–
Sorghum shell	5.00	–
The Mixed Silage	0.00	50.00
Corn	34.00	34.00
Soybean meal	7.00	5.50
Bran	7.50	8.00
Corn gluten meal	–	1.00
NaHCO_3_	0.50	0.50
Premix contained^2^	0.50	0.50
Salt	0.50	0.50
Total	100.00	100
Nutrient composition (% of DM)		
Digestive energy/DE (MJ/Kg)^3^	13.52	14.73
CP (%)	15.08	15.11
Ash (%)	4.36	12.33
NDF (%)	47.64	48.23
ADF (%)	23.71	27.17
Ca (%)	0.48	0.45
P (%)	0.38	0.39

1 Control: Based on peanut seedling, corn husk and sorghum shell for roughage; Mixed Silage: Based on the mixed silage for roughage.

2 Premix contained (per kg): VA 80 kIU, VD 25 kIU, VE 130 kIU, Fe 0.6 g, Mn 0.7 g, Zn 2.3 g, Cu 0.2 g, Se 8 mg, Ca 10%, P 1%, NaCl 10%.

3 DE was estimated according to NRC (2007). The others were measured values.

### Determination of growth performance

We recorded the fasting weight of each sheep before the formal start and end of morning feeding, that is, the initial weight (IW) and final weight (FW), to calculate the average daily gain (ADG). The amount of feed and leftovers fed to each sheep was recorded during the experiment to calculate the mean daily feed intake (DMI). Finally, the feed-to-weight ratio (F/W) was calculated according to ADG and DMI.


ADG=(FW−IW)/28

$$\begin{array}{c} \mathrm{DMI}=\left(\mathrm{feeding amount}\times \mathrm{DM}\;\mathrm{content}\right)\\ -\left(\mathrm{residual feed amount}\times \mathrm{residual feed}\;\mathrm{DM}\;\mathrm{content}\right) \end{array}$$

F/W=DMI/ADG

### Sampling

Before morning feed, 5 Hu sheep were randomly selected from the experimental group and the control group for slaughter after fasting for 24 h and having no access to water for 2 h. The sheep were euthanized by injecting thiopental sodium (0.125 mg/kg BW) and potassium chloride (5–10 ml), and then immediately bloodletting. After slaughtering, the rumen fluid was collected. About 50 ml of rumen fluid from each sheep was collected and filtered with four layers of gauze, and the pH value was measured by a pH meter (Mettler Toledo, Switzerland) immediately. Then, it was sub-packed into four 15 ml centrifuge tubes and stored at −80°C for analysis. Rumen fluid for VFA determination requires the addition of deproteinized solution (100 g metaphosphoric acid and 0.6 g crotonic acid per L) in a 1:1 ratio. During slaughter, we immediately collected blood, muscle (longissimus dorsi), rumen, kidney, spleen, liver, and small intestine (duodenum, jejunum, ileum) samples of Hu sheep.

### Determination of rumen fermentation parameters

The content of NH3-N in rumen fluid was determined by phenol sodium hypochlorite colorimetry (Broderick and Kang, 1980). Microprotein (MCP) content was determined by the trichloroacetic acid method (Cotta and Russell, 1982). The content of VFA in rumen fluid was determined by gas chromatography (GC-14B, Kyoto, Japan) meteorological chromatography internal standard method (Pan et al., 2016), and the TVFA and the ratio of acetate to propionate were calculated. The instruments and conditions of gas chromatography were the same as Zhang’s method (Zhang et al., 2021).

### Determination of antioxidant enzymes

The levels of superoxide dismutase (SOD), total antioxidant capacity (T-AOC), GSH-Px, malondialdehyde (MDA) and Catalase (CAT) in blood, muscle (longissimus dorsi), rumen, kidney, spleen, liver, and small intestine (duodenum, jejunum and ileum) samples were measured. The kit for index determination was purchased from Nanjing Jiancheng Biotechnology Co., Ltd., Nanjing, China.

### DNA extraction, PCR amplification, and computer

After the rumen fluid samples were thawed, the total microbial DNA of the rumen fluid was extracted using the HiPure Soil DNA Kit (purchased from D3142B, Magen Biotechnology Co., Ltd., GuangZhou, China). The extracted DNA was tested for purity and concentration (OD260/280 and OD260/230) with an ultra-micro spectrophotometer (NzanoDrop-1000, Thermo Fisher Scientific Co., Ltd., United States), and then 1% agarose gel electrophoresis was used.

Amplification was performed using bacterial V3-V4 variable region universal primers 341F (5′-CCTACGGGNGGCWGCAG-3′), 806R (5′-GGACTACHVGGGTWTCTAAT-3′). PCR used 2× Phanta Master Mix, 30 μl reaction system: 10 ng Genomic DNA, 15 μl 2 × Phanta Master Mix, 1 μl Bar-PCR primer F (10 μM), 1 μl Primer R (10 μM), ddH_2_O supplemented to 30 μl. Reaction program: initial denaturation at 95°C for 5 min; 27 cycles of denaturation at 95°C for 30 s, annealing at 55°C for 30 s, and elongation at 72°C for 45 s; stretch for 10 min at 72°C. After PCR amplification, the obtained products were detected by 2% gel electrophoresis. The PCR products with correct band size and appropriate concentration were sequenced on the computer. The sequencing company was Genepioneer Biotechnologies Co., Ltd (Nanjing, China) and the platform was Illumina miseq.

### Data analysis

We used SPSS Statistics V20.0 Software to test the normal distribution and homogeneity of the data. Vitamin content (V_A_, V_B2_, V_C_ and V_E_) and rumen fermentation parameters (pH, VFA, MCP and NH_3_-N) were tested in SPSS using independent samples T-test.

Species richness (Chao1 index) and diversity (Simpson and Shannon index) were calculated with the R language picante package and the difference test was performed with Mann–Whitney U nonparametric rank-sum test using IBM SPSS Statistics 20.0 software. Venn diagrams and Principal coordinate analysis (PCoA) diagrams were drawn through the ggplot2 package of the R language. At the same time, the similarity analysis (ANOSIM) calculated by the vegan package of R language shows the similarity between groups, where 0 = indistinguishable, 1 = identical. Determination of ANOSIM’s *p*-value based on 999 permutation tests. Canonical correlation analysis (CCA) was used to analyze the influence of environmental factors on microorganisms, which was performed through the vegan and ggrepel packages and visualized with ggplot2. Only those bacterial taxa with an abundance >0.1% in at least one sample were analyzed. KEGG microbial gene function prediction analysis was performed using PICRUSt according to previous protocol (Zhang et al., 2022). The Pearson correlations between differential flora and rumen fermentation parameters, as well as differential flora and differential metabolic pathways, were completed by the complot package in R language. When the correlation coefficient (R) was >|0.65| and the *p*-value was <0.05, the bacterial community and rumen fermentation parameters were considered to be significantly correlated (Wang et al., 2016).

## Results

### Vitamin content of roughage

In Table 2, the contents of V_B2_ and V_C_ in the mixed silage group were higher than those in the control group (*p* < 0.05), and there was no difference in the contents of V_A_ and V_E_ (*p* > 0.05).

**Table 2:** Comparison of vitamin contents between mixed silage and control.

	Groups			
Vitamin, μg/L	Control	Mixed silage	SEM	Value of *p*
V_A_	72.54	75.95	1.01	0.089
V_B2_	3.69^b^	4.10^a^	0.10	0.028
V_C_	23.23^b^	27.85^a^	0.94	0.003
V_E_	8.16	7.70	0.15	0.114

In the same row, values with no letter or the same letter superscripts mean no significant difference (*p* ≥ 0.05), while with different small letter superscripts mean significant difference (*p* < 0.05).

### Effects of mixed silage on growth performance of Hu sheep

**Figure 1:**
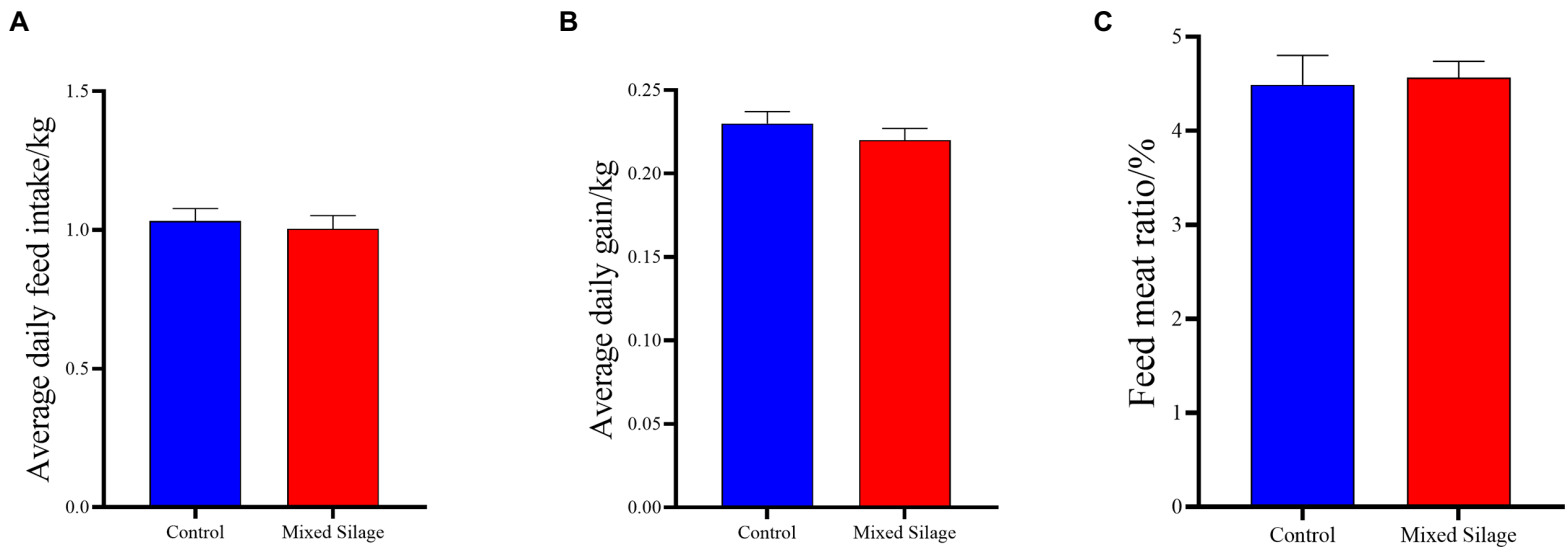
Effects of the mixed silage on growth performance of Hu sheep. **(A)** Average daily feed intake; **(B)** Average daily gain; **(C)** Feed meat ratio. Control: Based on peanut seedling, corn husk and sorghum shell for roughage in the diet; Mixed Silage: Based on the mixed silage for roughage in the diet.

It can be seen from Figure 1 that the mixed silage did not affect the DMI (Figure 1A), ADG (Figure 1B) and F/W (Figure 1C) of Hu sheep (*p* > 0.05).

### Effects of mixed silage on antioxidant properties of Hu sheep

**Figure 2:**
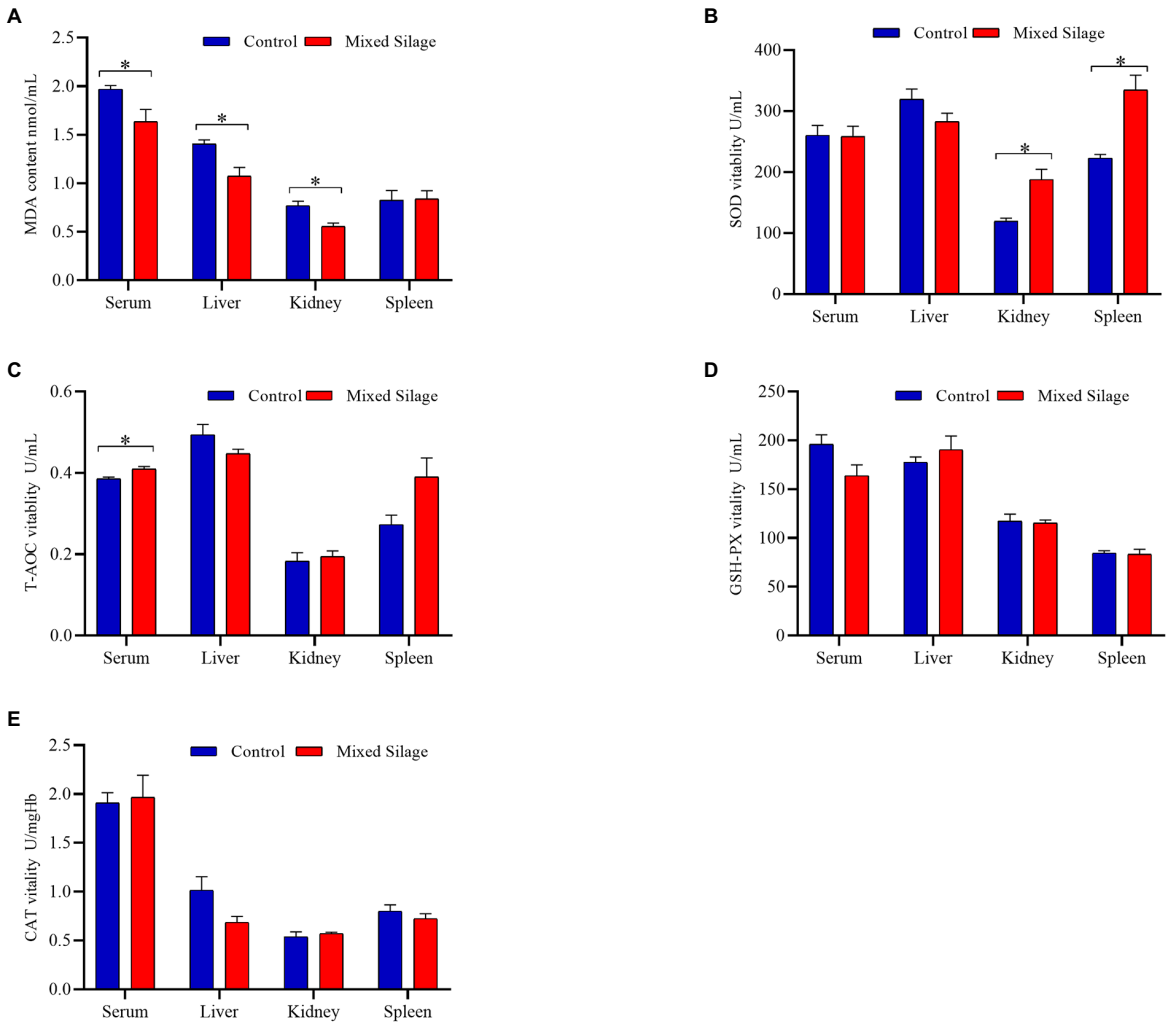
Effects of the mixed silage on antioxidant capacity of Hu sheep. **(A)** MDA content. **(B)** SOD vitality. **(C)** T-AOC vitality. **(D)** GSH-PX vitality. **(E)** CAT vitality. Control: Based on peanut seedling, corn husk and sorghum shell for roughage in the diet; Mixed Silage: Based on the mixed silage for roughage in the diet. “*” means indicates a significant difference (*p* < 0.05). No “*” indicates that the difference is not significant (*p* > 0.05). The same as below.

**Figure 3:**
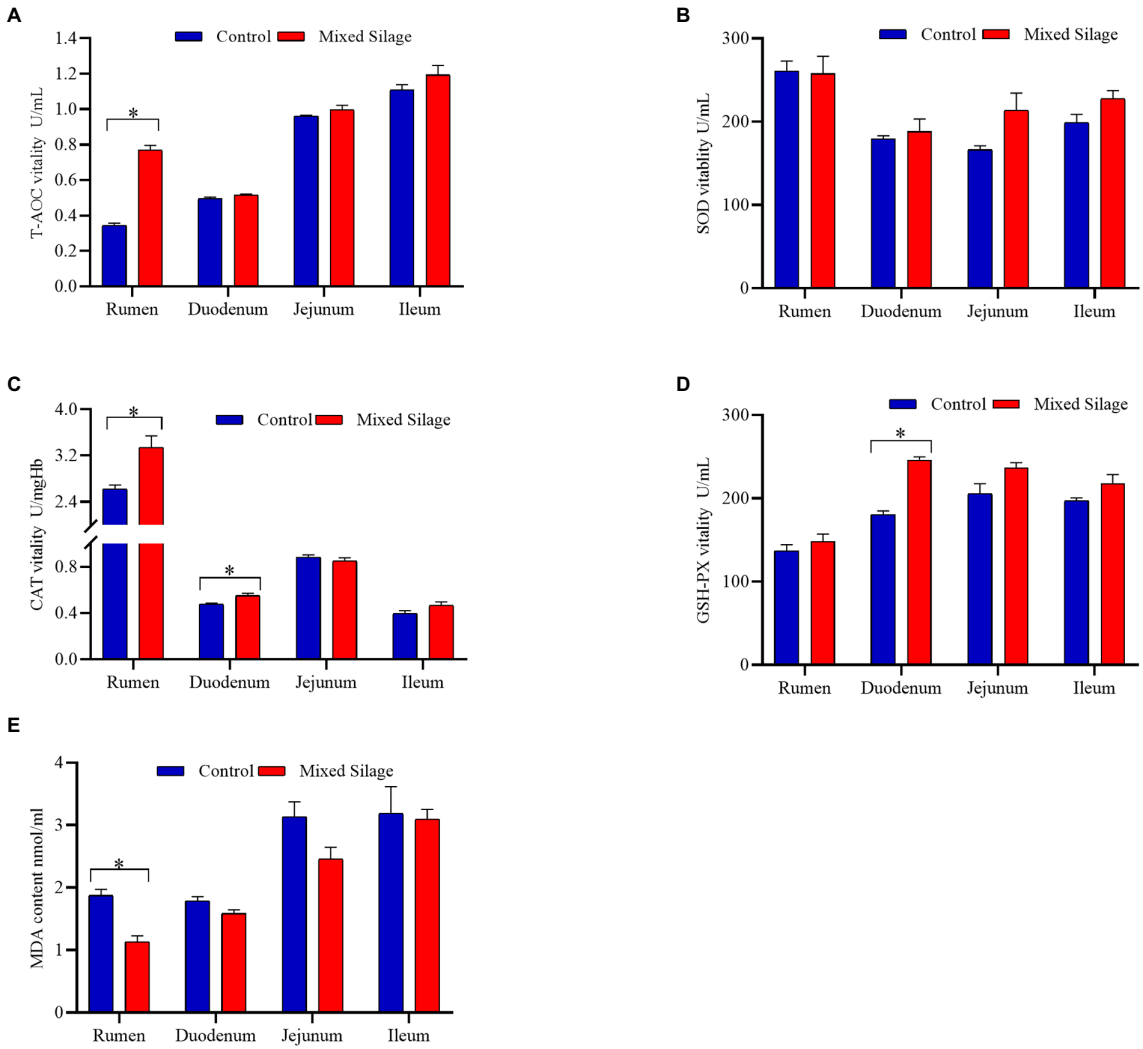
Effects of the mixed silage on antioxidant capacity in the digestive tract of fattening Hu sheep. **(A)** T-AOC vitality. **(B)** SOD vitality. **(C)** CAT vitality. **(D)** GSH-PX vitality. **(E)** MDA content. Control: Based on peanut seedling, corn husk and sorghum shell for roughage in the diet; Mixed Silage: Based on the mixed silage for roughage in the diet.

Figure 2 shows the comparison of antioxidant properties of blood and organs between mixed silage group and control group. Compared with the control group, the mixed silage increased the concentration of T-AOC in serum and SOD in kidney (*p* < 0.05). In addition, the content of MDA in serum, liver and kidney decreased (*p* < 0.05). The effects of the mixed silage on intestinal antioxidant performance of Hu sheep is shown in Figure 3. The mixed silage could increase the content of T-AOC and CAT in rumen and duodenum. In addition, it could reduce the content of MDA in rumen (*p* < 0.05). The results showed (Table 3) that the content of MDA in the mixed silage group was lower (*p* < 0.05), while the antioxidant indexes such as T-AOC, SOD, GSH-Px, and CAT were higher than those in the control groups with no differences (*p* > 0.05).

**Table 3:** Effects of the mixed silage on muscle anti-oxidation of Hu sheep.

Item	Groups^1^		SEM^2^	Value of *p*
	Control	Mixed silage		
T-AOC, U/mg	1.39	1.50	0.07	0.477
SOD, U/mg	200.73	206.58	5.02	0.591
GSH-Px, U/mg	138.90	140.24	3.48	0.861
CAT, U/mg	1.32	1.48	0.05	0.095
MDA, mmol/mg	5.01^a^	3.83^b^	0.28	0.022

^1^Control: Based on peanut seedling, corn husk and sorghum shell for roughage; Mixed Silage: Based on the mixed silage for roughage.

^2^SEM, standard error of the mean.

^a,b^In the same row, values with no letter or the same letter superscripts mean no significant difference (*p* ≥ 0.05), while with different small letter superscripts mean significant difference (*p* < 0.05).

### Rumen fermentation parameters

The results in the Table 4 showed that the mixed silage could reduce the pH and the concentrations of isovalerate and isobutyrate (*p* < 0.05). In addition, it increased the concentrations of TVFA, NH_3_-N, butyrate, and valerate in rumen fluid (*p* < 0.05).

**Table 4:** Effects of the mixed silage on rumen fermentation parameters in Hu sheep.

Item	Groups^1^		SEM^2^	Value of *p*
	Control	Mixed silage		
Ruminal pH	7.09^a^	6.91^b^	0.04	0.019
NH_3_-N (mg/dL)	7.38^b^	11.73^a^	0.86	0.002
MCP (mg/dL)^3^	19.24	18.71	0.35	0.479
VFAs^4^				
TVFA (mmol/L)^5^	68.77^b^	78.23^a^	1.93	0.004
Acetate (mmol/L)	43.31	45.08	0.80	0.293
Propionate (mmol/L)	17.39	18.82	0.88	0.448
Isobutyrate (mmol/L)	1.13^a^	0.88^b^	0.07	0.049
Butyrate (mmol/L)	4.43^b^	8.62^a^	0.83	0.002
Isovalerate (mmol/L)	1.98^a^	1.14^b^	0.19	0.012
Valerate (mmol/L)	0.53^b^	1.11^a^	0.12	0.005
A:P^6^	2.51	2.57	0.14	0.850

1 Control: Based on peanut seedling, corn husk and sorghum shell for roughage; Mixed Silage: Based on the mixed silage for roughage.

2 SEM, standard error of the mean.

3 MCP, Microbial protein.

4 VFAs, volatile fatty acids.

5 TVFA, total volatile fatty acids.

6 A:P, Ratio of acetic acid to propionic acid.

^a,b^In the same row, values with no letter or the same letter superscripts mean no significant difference (*p* ≥ 0.05), while with different small letter superscripts mean significant difference (*p* < 0.05).

### Rumen microbial diversity, richness, and structure

**Figure 4:**
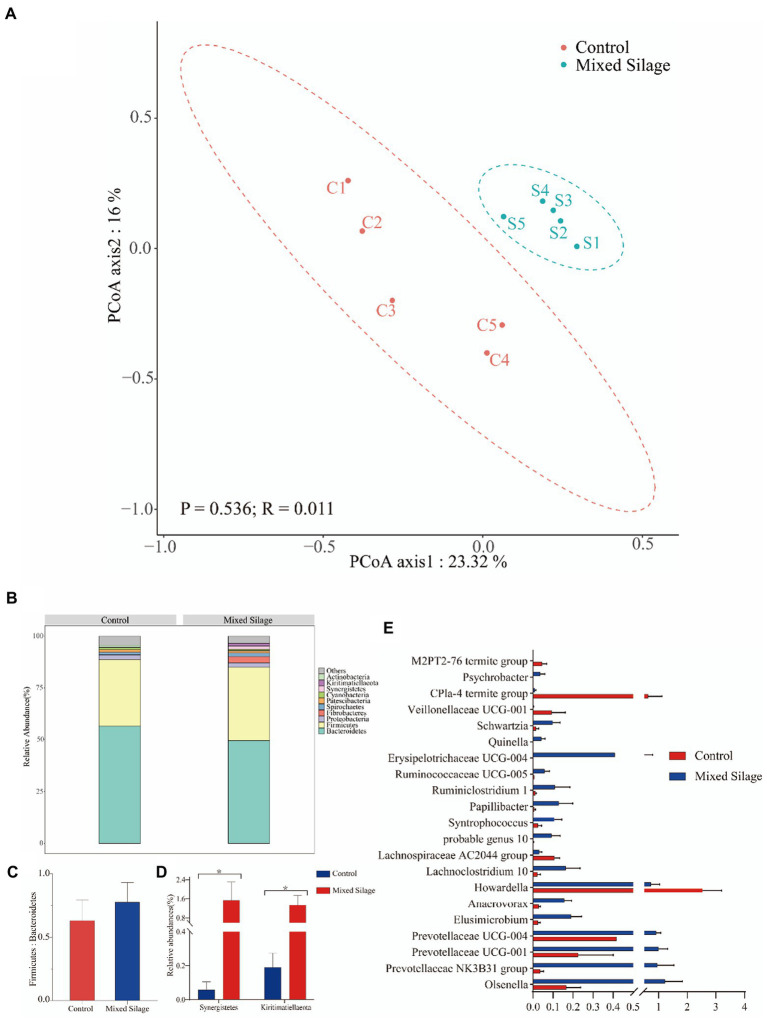
Structure of rumen bacterial flora in Hu Sheep. **(A)** Principal coordinate analysis (PCoA) of the bacterial community structure of ruminal microbiota in the control and mixed silage groups. **(B)** The composition of the top 10 phyla in rumen fluid. **(C)** Ratio of the proportion of phyla Firmicutes to the proportion of Bacteroidetes. **(D)** The phyla with the difference in the top 10 phyla. **(E)** The proportion of the bacterial genus with significant differences among the two groups. Generas with a proportion >0.1% in any one sample will be analyzed. Control: Based on peanut seedling, corn husk and sorghum shell for roughage in the diet; Mixed Silage: Based on the mixed silage for roughage in the diet. PCoA plots were constructed using Bray–Curits distance.

The results in the Supplementary Table 1 showed that the OTU, Chao1 index, Shannon index and Simpson index of the silage group were higher than those of the control group (*p* < 0.05). It can be seen from the species accumulation curve (Supplementary Figure 1A) that when the number of samples taken reaches 8, the detection rate of new species is further slowed down, and the species accumulation curve becomes flat (close to the horizontal state), indicating that adding new sequencing samples was important for the discovery of rumen. The contribution of new microbial species was getting smaller and smaller, so the sequencing samples in this experiment can completely cover most of the microorganisms in the rumen environment of Hu sheep. Clustering of sequences with 97% similarity revealed that the two groups shared 1,187 OTUs, 348 were shared by the silage group, and 172 were exclusively shared by the control group (Supplementary Figure 1B). PCoA based on Bray–Curtis distance was drawn for comparison of the two treatments. As it can be seen from Figure 4A that the samples of the mixed silage group were completely separated from the samples of the control group. The results of ANOSIM analysis showed that mixed silage could affect the rumen microflora structure of the fattening Hu sheep (*R* = 0.536, *p* = 0.011).

At the phyla level, Bacteroidetes and Firmicumtes were the dominant phyla in both the mixed silage group and the control group (Figure 4B). Furthermore, the ratio of Firmicutes to Bacteroidetes was not different between the two groups (*p* > 0.05; Figure 4C). Compared with the control group, the mixed silage group increased the proportion of Synergistetes and Kiritimatiellaeota in rumen fluid (*p* < 0.05; Figure 4D). At the genera level, 21 genera (relative abundance of more than 0.1%) were different between the two groups (*p* < 0.05; Figure 4E). Compared with the control group, the mixed silage group increased the proportion of *Olsenella*, *Prevotellaceae NK3B31 group*, *Prevotellaceae UCG-001*, *Prevotellaceae UCG-004*, *Elusimicrobium*, *Anaerovorax*, *Lachnoclostridium 10*, *probable genus 10*, *Papillibacter*, *Syntrophococcus*, *Erysipelotrichaceae*, *Ruminiclostridium 1*, *Ruminococcaceae UCG-005*, *Quinella*, *Schwartzia*, *Veillonellaceae UCG 001* and *Psychrobacter* (*p* < 0.05), but decreased the proportion of *Howardella*, *Lachnospiraceae AC2044 group*, *Veillonellaceae UCG 001*, *CPla 4 termite group* and *M2PT2 76 termite group* relative abundance (*p* < 0.05).

### CCA of rumen microflora and differential rumen fermentation parameters

**Figure 5:**
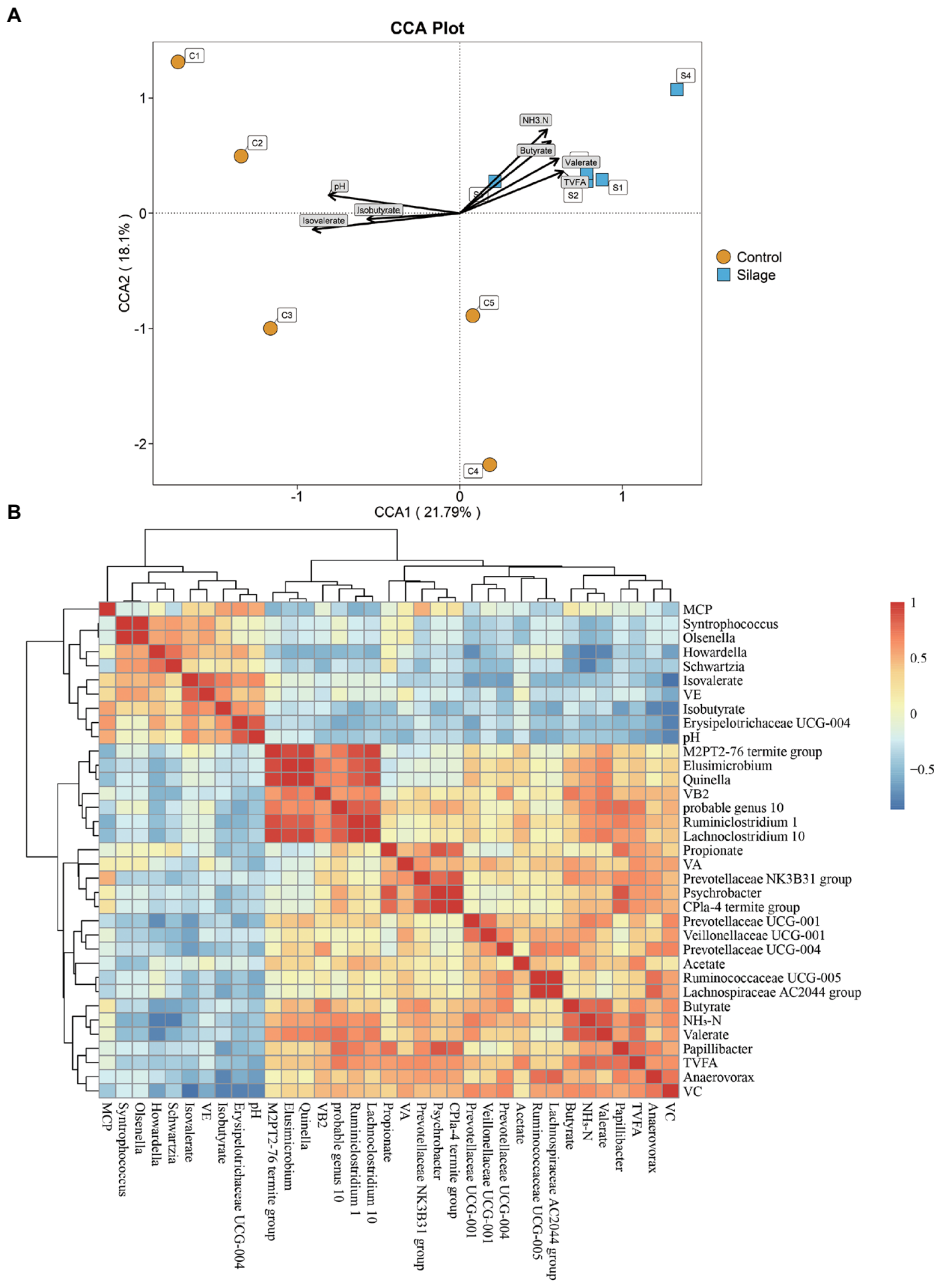
Correlation results between rumen microbiota structure and fermentation parameters or vitamins. **(A)** CCA to prove the associations between the rumen fermentation parameters and the bacterial community structure. The length of an arrow expresses the relative influence of its corresponding rumen fermentation parameters on the distribution of the bacterial community analyzed. Centroid is displayed for each treatment. The samples are represented by circles and square symbols, respectively. **(B)** Correlation analyses between proportion of differential bacteria genera and ruminal fermentation parameters and vitamins.

The relationship between the rumen bacterial community and rumen fermentation parameters were analyzed by Canonical correspondence analysis (CCA). As shown in Figure 5A, the relationship between the rumen microflora and isovalerate (*p* = 0.002), NH_3_-N (*p* = 0.003), butyrate (*p* = 0.021), pH (*p* = 0.022), valerate (*p* = 0.048) and TVFA (*p* = 0.064) contributed to the flora.

### Correlation analysis of microorganisms, vitamins and fermentation parameters

As shown in Figure 5B, there was a negative correlation between pH and *Anaerovorax*, and a positive correlation with *Erysipelotrichaceae UCG-004* (*p* < 0.05). NH_3_-N showed a positive correlation with *Prevotellaceae UCG-001*, *Lachnoclostridium 10* and *probable genus 10*, but a negative correlation with *Schwartzia* and *Howardella* (*p* < 0.05). There was a positive correlation between TVFA and *Prevotellaceae NK3B31 group*, *Papillibacter* and *probable genus 10* (*p* < 0.05). Propionate showed a positive correlation with *Psychrobacter*, *Papillibacte* and *CPla-4 termite group* (*p* < 0.05). Isobutyrate showed a negative correlation with *Anaerovorax* and *Papillibacter*. Butyrate showed a positive correlation with *Prevotellaceae NK3B31* and *Prevotellaceae UCG 004* (*p* < 0.05). Valerate showed a positive correlation with *Quinella*, *Elusimicrobium*, *Lachnoclostridium 10* and *probable genus 10* (*p* < 0.05). V_B2_ was positively correlated with *Elusimicrobium*, *Quinella*, and *Lachnoclostridium 10* (*p* < 0.05). V_C_ was positively correlated with *Anaerovorax*, *Prevotellaceae UCG-004* and *Prevotellaceae UCG-001*, but negatively correlated with *Erysipelotrichaceae UCG-004* (*p* < 0.05).

### Prediction of rumen bacterial function

**Figure 6:**
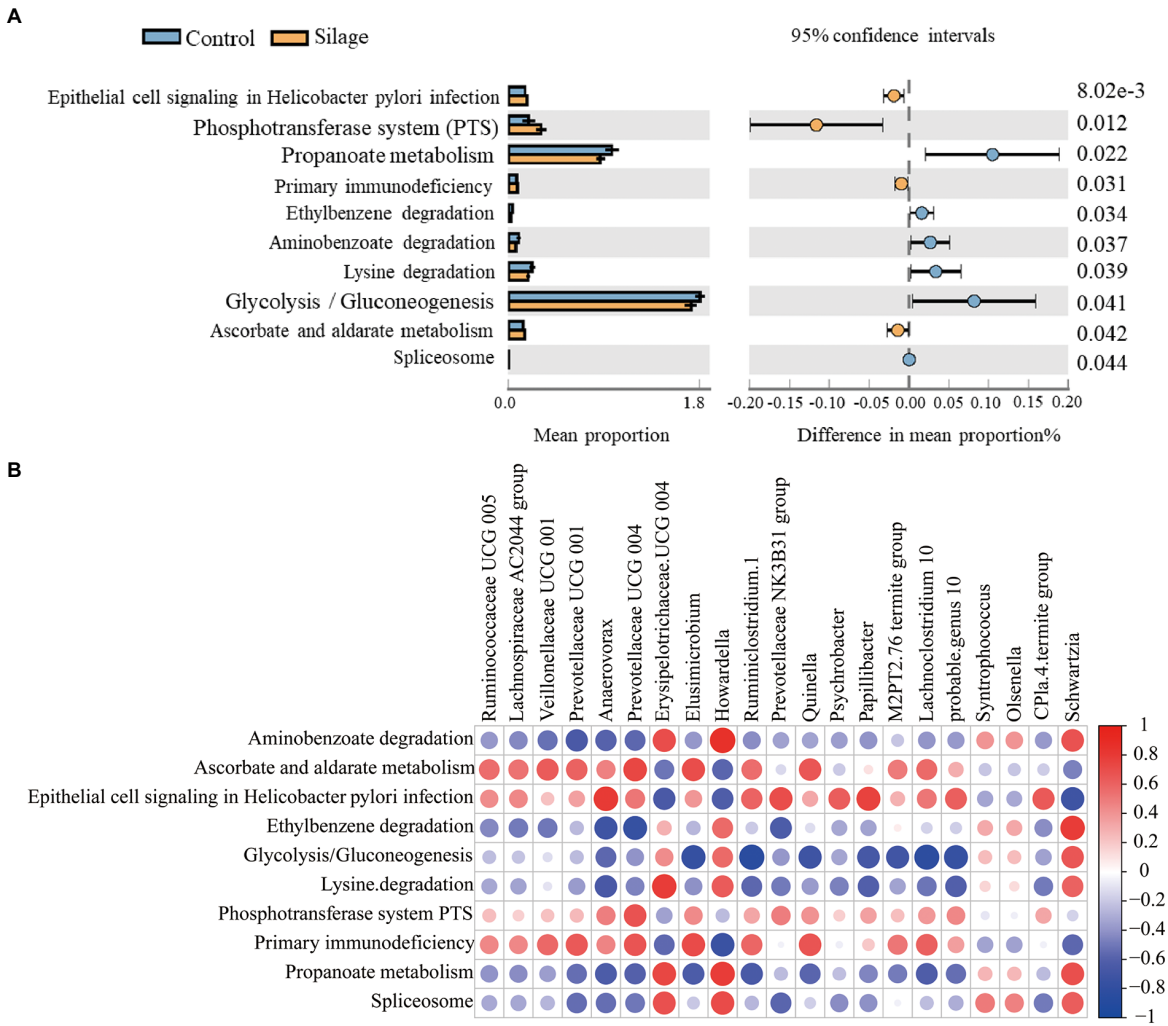
Prediction of metabolic pathways and their correlation with rumen microbes. **(A)** Analysis of differences in functional prediction pathways of rumen flora between the control group and the mixed silage group. **(B)** Correlation analysis between differential microorganisms and differential pathways. In this graph, each row represents differential metabolic pathways and each column represents differential microorganisms at the genera level. Red circles indicate positive correlations, and blue circles indicate negative correlations.

There were 10 significantly different metabolic pathways in the microbiota prediction function between the mixed silage group and the control group (Figure 6A). The mixed silage up-regulated epithelial cell signaling in helicobacter pylori infection, phosphotransferase system (PTS), primary immunodeficiency and ascorbate and aldarate metabolism (*p* < 0.05), while downregulated aminobenzoate degradation, ethylbenzene degradation, glycolysis/gluconeogenesis, lysine degradation, propanoate metabolism and spliceosome metabolic pathways (*p* < 0.05).

### Correlation analysis between microorganisms and pathways

As shown in Figure 6B, aminobenzoate degradation had a positive correlation with *Erysipelotrichaceae UCG-004*, *Howardella* and *Schwartzia*, and a negative correlation with *Prevotellaceae UCG-001* (*p* < 0.05). Ascorbate and aldarate metabolism were positively correlated with *Veillonellaceae UCG-001*, *Prevotellaceae UCG-004*, *Elusimicrobium* and *Quinella* (*p* < 0.05). Epithelial cell signaling in helicobacter pylori infection was positively correlated with *Anaerovorax*, *Prevotellaceae NK3B31 group*, *Papillibacter* and *CPla-4 termite group*, while negatively correlated with *Erysipelotrichaceae UCG-004* and *Schwartzia* (*p* < 0.05). Ethylbenzene degradation showed a positive correlation with *Schwartzia* and a negative correlation with *Anaerovorax* and *Prevotellaceae UCG-004* (*p* < 0.05). Glycolysis/gluconeogenesis showed a positive correlation with *Schwartzia*, and a negative correlation with *Elusimicrobium*, *Ruminiclostridium 1*, *Quinella*, *Papillibacter*, *M2PT2–76 termite group*, *Lachnoclostridium 10*, and *probable genus 10* (*p* < 0.05). Lysine degradation had a positive correlation with *Erysipelotrichaceae UCG-004* and *Howardella*, while a negative correlation with *Anaerovorax* (*p* < 0.05). Phosphotransferase system (PTS) had a positive correlation with *Prevotellaceae UCG-004* (*p* < 0.05). Primary immunodeficiency was positively correlated with *Prevotellaceae UCG-001*, *Prevotellaceae UCG-004* and *Elusimicrobium*, but negatively correlated with *Quinella* (*p* < 0.05). Spliceosome had a positive correlation with *Erysipelotrichaceae UCG-004* and *Howardella* (*p* < 0.05). In addition, propanoate metabolism was found negatively correlated with *Ruminiclostridium 1* (*p* < 0.05).

## Discussion

The production, utilization and elimination of free radicals in the body are in a dynamic balance. When this balance is broken, the body produces oxidative stress, the reactive oxygen species cause lipid peroxidation, giving rise MDA as a final product (Honaramooz et al., 2011). The complex antioxidant system in the body is influenced by dietary intake of antioxidants (taurine, etc.), vitamins (V_C_ and V_E_), and minerals (Zn etc.; Agarwal and Gupta, 2005). SOD is an intracellular antioxidant enzyme that protects against oxidative stress (Bhatia et al., 2003). CAT exists in red blood cells and peroxides in certain tissues, and can make H_2_O_2_ react to generate water and oxygen molecules (Zhao et al., 2017). Serum T-AOC is primarily considered to be a proxy for the balance between oxidative and antioxidant compounds in the body, and it provides more biologically relevant information than any other individual antioxidants (Ghiselli et al., 2000). The results showed that T-AOC in serum and rumen of the mixed silage group were significantly higher than those in the control group. SOD activity in the kidney was observed significantly higher, while CAT activity in the rumen and duodenum was seen higher significantly. In addition, MDA content in the muscle, serum, liver, kidney and rumen of the mixed silage group was significantly lower than that of the control group. This may be caused by the higher content of V_C_ in the mixed silage. Sahoo et al. (2021) results showed that the addition of fresh fruit and vegetable tails to the diet increased the ability of T-AOC in the blood of ewes. V_C_ can be used to maintain the oxidation state of cells and remove other oxidizing substances, so as to improve the T-AOC of cells (Sordillo and Aitken, 2009). A study by Seifzadeh et al. (2022) also showed that by adding 20 mg of V_C_ to prenatal dairy cow diets significantly reduced blood MDA concentration. By adding 50 mg/ml V_C_ to drinking water can increase the level of T-AOC in the myocardium and serum of chickens, and improves its antioxidant capacity (Yin et al., 2020). In addition, by adding V_C_ to the diet can also increase the activity of MnSOD (a kind of SOD) in chicken liver and kidney (Ozturk-Urek et al., 2001).

Partovi et al. (2020) fed Fasander lambs with a mixed diet of broccoli by-products and wheat straw (ratio 69:31) and found that the mixed diet did not affect the rumen fermentation of lambs. In our experiment, the mixed silage increased the concentrations of TVFA, NH_3_-N and butyric acid in the rumen fluid, while the pH of the rumen fluid decreased. The normal range of rumen pH is 5.5–7.0, but it may also reach to 7.5 (Kim et al., 2012; Anantasook et al., 2013). The pH of the rumen in the two groups of diets was within the normal range, whereas the pH of the mixed silage group was lower than that of the control group, which may be caused by the increase of TVFA. The NH_3_-N in the rumen originates from the degradation of endogenous nitrogenous substances or exogenous nitrogenous substances, and is also the main substance for the synthesis of MCP, which can reflect the utilization of nitrogenous substances by rumen microorganisms to a certain extent. All animals in both groups had NH_3_-N concentrations in the rumen above 5 mg/dl, which is the minimum concentration required to ensure optimal growth of rumen microorganisms (Satter and Slyter, 1974). The differences in NH_3_-N in the two groups of diets indicated that the mixed silage could affect the release of N in the rumen and the efficiency of MCP synthesis after being absorbed by microorganisms. The increase of VFA content in the mixed silage group may be caused by the increase of rumen microbial reproduction and activity. VFAs which account for 75% of the total energy requirements provided by ruminants, are the main product of rumen microbial fermentation, and are critical to the overall metabolism of ruminants (Bergman, 1990; Dijkstra, 1994). Butyrate can effectively stimulate the proliferation and growth of the rumen epithelium (Gorka et al., 2018). The increase of butyric acid in the mixed silage group in this experiment may be due to the increase of butyric acid-producing bacteria in the rumen.

The rumen microbes are complex and diverse, and play an important role in the digestion of nutrients and the functioning of the rumen. Diet type, structure and feeding method can significantly affect the species and quantity of ruminant microorganisms in ruminants (van Lingen et al., 2016). The mixed silage increased the species diversity and richness of rumen microorganisms, which may be due to the higher metabolizable and digestible energies of the silage group, promoted the growth and reproduction of microorganisms, and improved the richness and diversity of microorganisms. The results of PCoA and ANOSIM clearly showed that the mixed silage significantly improved the rumen microbial composition. Additionally, CCA analysis showed that among the different rumen fermentation parameters, isovalerate, NH_3_-N, butyrate, pH, valerate and TVFA had the greatest influence on rumen microorganisms.

The ecosystem of rumen microorganisms is dominated by its core flora which is not affected by the nutrients from feed sources (Petri et al., 2012; Henderson et al., 2015). Bacteroidea and Firmicutes are two dominant microflora in the structure of rumen microbial flora (Singh et al., 2012; Pitta et al., 2014). Previous studies have shown that the main function of Bacteroidea members is to hydrolyze proteins and degrade carbohydrates, while Firmicutes members are mainly involved in energy utilization (Wu et al., 2011; Chen et al., 2015). The ratio of Firmicutes to Bacteroidetes is generally considered to be related to the metabolic potential of the intestinal microbiota and can regulate the metabolism, physiology and health of the host (Tilg and Kaser, 2011; Clemente et al., 2012). Our results showed that the mixed silage did not have a significant effect on Firmicutes, Bacteroidetes or their ratios, which is consistent with previous studies (Zhang et al., 2014). Notably, at the level of the top 10 phyla in relative abundance, the mixed silage increased the proportion of Synergistetes and Kiritimatiellaeota. Synergistetes is part of the gut microbiota of healthy animals and can degrade amino acids (Leser et al., 2002; Menes and Muxi, 2002; Godon et al., 2005). Studies have pointed out that Synergistetes isolated from sheep rumen can not only utilize arginine and histidine to improve nitrogen utilization, but also degrade toxins present in feed (McSweeny et al., 1993; Jumas-Bilak et al., 2009). Kiritimatiellaeota is a supergroup of the planktonic Chlamydia verrucobacterium (PVC) whose primary function is to break down complex polysaccharides and glycoproteins (Spring et al., 2016; Mu et al., 2020).

The experimental results showed that the proportions of *Prevotellaceae NK3B31 group*, *Prevotellaceae UCG-001* and *Prevotellaceae UCG-004* were increased in the mixed silage group. *Prevotellaceae UCG-001*, *Prevotellaceae UCG-004* and *Prevotellaceae NK3B31 group,* all belong to the Prevotellaceae family. The results of association analysis showed that *Prevotellaceae UCG-004* was positively correlated with Vc, ascorbate and aldarate metabolism. In mammals, V_C_ is abundant and an effective water-soluble antioxidant for reducing oxidative stress, but its impact on the rumen ecology is unknown (Ghanem et al., 2008; Matsui, 2012). The use of antioxidants to improve animal health and product quality has received increasing attention (Schelling et al., 1995). The basic physiological needs of the organism are thought to be met by L-ascorbic acid (or V_C_) generated in the liver of ruminants (Ranjan et al., 2012). Compared to other animals, ruminants may require vitamin supplementation during feeding because microbes in the rumen completely destroy V_C_ in the feed (Kim et al., 2012; Ranjan et al., 2012). Another *in vitro* study has shown that high doses of V_C_ have a certain positive effect on rumen microorganisms (Tagliapietra et al., 2013). Therefore, it is speculated that the higher V_C_ content in the mixed silage group could increase the proportion of *Prevotellaceae UCG-004*, but it remains to be studied. Prevotellaceae is positively correlated with the content of ADF and NDF in the diet (An et al., 2020). In this experiment, the ADF content in the mixed silage group was higher, which may be another reason for the increase in the relative abundance of *Prevotellaceae UCG-004*. *Prevotellaceae UCG-004* can ferment carbohydrates and produce SCFAs including acetate and butyrate. The process of butyrate metabolism is linked to the interconversion of “pentose and glucuronic acid,” and the “ascorbate and aldarate metabolism” through dehydrogenases together (Heinritz et al., 2016; Shi et al., 2020).

## Conclusion

In summary, the mixed silage could improve rumen fermentation and enhance the antioxidant capacity of Hu sheep. In this experiment, the mixed silage also increased the concentrations of TVFA, NH_3_-N and butyric acid in the rumen, whereas the pH of the rumen fluid was reduced. In addition, the mixed silage increased the activity of T-AOC in blood, SOD in liver and kidney, and decreased the content of MDA in blood. The improvement of the antioxidant capacity of Hu sheep may be directly caused by the higher V_C_ content in the mixed silage, or it may be caused by the up-regulation of ascorbate and aldarate metabolism in Hu sheep. The upregulation of ascorbate and aldarate metabolism may be related to the increased proportion of *Prevotellaceae UCG-004* caused by higher V_C_ or ADF content in the mixed silage.

## Data availability statement

The datasets provided in this study can be found in the online repository. The name and login number of the repository are as follows: NCBI SRA Bioproject, login number: PRJNA795847.

## Ethics statement

The animal study was reviewed and approved by Yangzhou Veterinary Animal Welfare Committee of the Ministry of agriculture of China [Yangzhou, China, license No. syxk (Su) 2019-0029]. Written informed consent was obtained from the owners for the participation of their animals in this study.

## Author contributions

MW and CL designed this study and wrote the manuscript of this study. CL, XZ, RQ, ZZ, ZL, YL, MZ, and XY conducted experiments and collected samples. Animal data were analyzed by CL, MZ, XZ, ZZ, YL, NC, and ZL. CL, KS, NC, WL, and MW performed bioinformatics analysis and visualization of data and draft manuscripts. All authors contributed to the article and approved the submitted version.

## Funding

This research was funded by the National 14th and 13th Five-Year Plan Key Research and Development Program (2021YFD1600702, 2018YFD0502100, 2017YFD0800200), the key program of State Key Laboratory of Sheep Genetic Improvement and Healthy Production (2021ZD07, NCG202232), and the Priority Academic Program Development of Jiangsu Higher Education Institutions (PAPD), China.

## Conflict of interest

The authors declare that the research was conducted in the absence of any commercial or financial relationships that could be construed as a potential conflict of interest.

## Publisher’s note

All claims expressed in this article are solely those of the authors and do not necessarily represent those of their affiliated organizations, or those of the publisher, the editors and the reviewers. Any product that may be evaluated in this article, or claim that may be made by its manufacturer, is not guaranteed or endorsed by the publisher.
