# Supplementary material for: Mixed silage with Chinese cabbage waste enhances antioxidant ability by increasing ascorbate and aldarate metabolism through rumen Prevotellaceae UCG-004 in Hu sheep
Source: Front Microbiol. 2022 Aug 26;13:978940. doi: 10.3389/fmicb.2022.978940 (PMC9459383; doi:10.3389/fmicb.2022.978940)
Supplement: Supplementary file 2 [file Data_Sheet_1.docx]

**Supplementary Table 1.** Number of observed species, richness and diversity indices in ruminal samples from control and mixed silage in Hu sheep.

| Item | Experimental treatment^1^ | | SEM^2^ | *P* -value |
| --- | --- | --- | --- | --- |
|  | Control | Mixed Silage |  |  |
| OTUs | 739.00^b^ | 1011.20^a^ | 52.209 | 0.009 |
| Chao1 index | 865.73^b^ | 1128.19^a^ | 48.508 | 0.009 |
| Shannon index | 6.18^b^ | 7.41^a^ | 0.240 | 0.009 |
| Simpson index | 0.96^b^ | 0.98^a^ | 0.005 | 0.028 |

Note: ^1^Control: Based on peanut seedling, corn husk and sorghum shell for roughage in the diet; Mixed Silage: Based on the mixed silage for roughage in the diet.

^2^standard error of the mean.


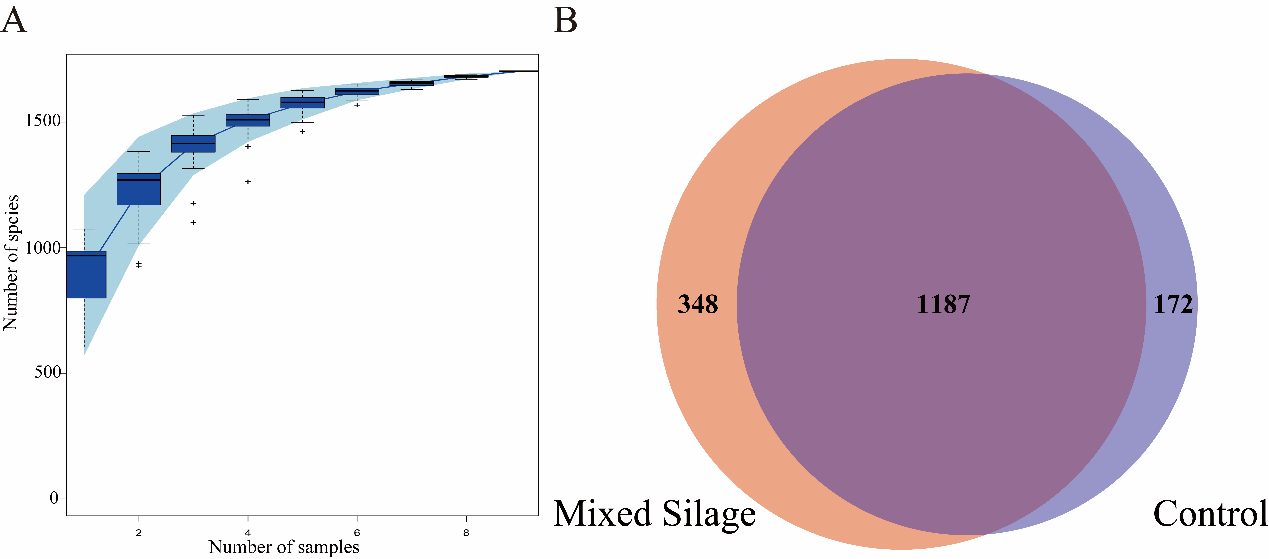


**Supplementary Figure 1.** Ruminal microbial OTUs with the different dietary groups. A) Species Accumulation (SA) analysis. B) Venn diagram of ruminal bacterial OTUs.

Note: Control, Control group; Mixed Silage, Mixed silage group; Control: Based on peanut seedling, corn husk and sorghum shell for roughage in the diet; Mixed Silage: Based on the mixed silage for roughage in the diet.
